# Enhanced Redox Cycle of Rod-Shaped MIL-88A/SnFe_2_O_4_@MXene Sheets for Fenton-like Degradation of Congo Red: Optimization and Mechanism

**DOI:** 10.3390/nano14010054

**Published:** 2023-12-24

**Authors:** Eman M. Abd El-Monaem, Nouf Al Harby, Mervette El Batouti, Abdelazeem S. Eltaweil

**Affiliations:** 1 Department of Chemistry, Faculty of Science, Alexandria University, Alexandria 21934, Egypt; emanabdelmonaem5925@yahoo.com (E.M.A.E.-M.); mervette.elbatouti@alexu.edu.eg (M.E.B.); abdelazeemeltaweil@alexu.edu.eg (A.S.E.); 2 Department of Chemistry, College of Science, Qassim University, Buraidah 51452, Saudi Arabia; 3 Department of Engineering, College of Engineering and Technology, University of Technology and Applied Sciences, Sultanate of Oman

**Keywords:** synergistic effect, redox reactions, Fenton-like reaction, MXene, mechanism

## Abstract

This study intended to fabricate a novel Fenton-like catalyst by supporting the rod-like MIL-88A and the magnetic tin ferrite nanoparticles (SnFe_2_O_4_) on the MXene sheets (MIL-88A/SnFe_2_O_4_@MXene). The well fabrication and determination of the MIL-88A/SnFe_2_O_4_@MXene properties were investigated using SEM, XPS, VSM, Zeta potential, XRD, and FTIR tools. The Fenton-like degradation reaction of CR by MIL-88A/SnFe_2_O_4_@MXene was thoroughly studied to identify the optimal proportions of the catalyst components, the impact of CR and H_2_O_2_ concentrations, as well as the effect of raising the temperature and the pH medium of the catalytic system and the catalyst dosage. Kinetics studies were executed to analyze the decomposition of CR and H_2_O_2_ using First-order and Second-order models. Furthermore, the degradation mechanism was proposed based on the scavenging test that proceeded in the presence of chloroform and t-butanol, in addition to the XPS analysis that clarified the participation of the containing metal species: Fe, Sn, and Ti, and the formation of a continual redox cycle. The obtained intermediates during the CR degradation were defined by GC–MS. A recyclability test was performed on MIL-88A/SnFe_2_O_4_@MXene during five runs of the Fenton-like degradation of CR molecules. Finally, the novel MIL-88A/SnFe_2_O_4_@MXene Fenton-like catalyst could be recommended as a propitious heterogeneous catalyst with a continuous redox cycle and a recyclability merit.

## 1. Introduction

Recently, the world has dug deep into water pollution, threatening the subsistence of freshwater. Undeniably, the excessive utilization of chemicals in varied industries leads to aggravating the water pollution affair. Since draining wastewater from industrial sectors contains many detrimental substances like dissolved metals, phenols, dyes, and polycyclic compounds, the vast jeopardy threatens the presence of adequate freshwater on the earth [1]. Particularly, synthetic dyes have innumerable applications, including plastic, leather, hair coloring, paper, textiles, cosmetics, and printing [2]. Hence, seventy thousand tons of about one hundred thousand types of dyes are produced annually, and the released percentage of dyes in water bodies ranges from 12 to 15% [3]. Unfortunately, these dyes are nondegradable and photostable, so they remain in the aquatic media for a prolonged duration.

Congo red (CR) is an azo dye, applied immensely in textile industries because of its high affinity to cellulose fibers [4]. Additionally, CR is used for amyloidosis diagnosing and as a pH indicator. Despite that, CR metabolizes to the carcinogenic benzidine and results in human diseases, such as respiratory and eye diseases, gastrointestinal irritation, and allergies [5]. The chemical structure complexity of CR endows it with superhigh stability in aquatic media and high degradation resistance [6]. Consequently, purification strategies have been instigated and progressed to conquer the hazard of CR, including precipitation, flocculation, biodegradation, adsorption, ozonation, and advanced oxidation processes (AOPs) [7,8,9,10].

The Fenton process is deemed to be one of the most creditable AOPs that exhibited auspicious application potential in degrading many detrimental contaminants. The Fenton process is distinguished by its excellent efficacy, acceptable cost, low toxicity, and simple operational features [11]. There are two types of Fenton reactions: the classical Homo-Fenton reaction proceeded by iron solution and hydrogen peroxide (H_2_O_2_) to form hydroxyl radicals (•OH), following the Weiss–Haber equation [5]. However, Homo-Fenton consumes large concentrations of iron species. Additionally, the degradation aptitude of the Homo-Fenton reaction is controlled by the pH medium of the catalytic system, clarifying a higher performance at a low acidic medium (pH < 3). Hence, it is essential to neutralize the purified water before throwing it out, which renders Homo-Fenton quite costly and monotonous [12]. The Hetero-Fenton reaction takes place in a solid/liquid interface since iron or any transition metal species are used in a solid phase, facilitating the catalyst separation and recyclability. Furthermore, the Hetero-Fenton reaction could proceed in a wide pH scale, which is one more merit over the Homo-Fenton reaction [13]. Consequently, countless substances have been developed to be efficient Hetero-Fenton catalysts, comprising metal–organic frameworks (MOFs), nanoparticles (NPs), carbon materials, MXene, clays, etc.

The unique characteristics of MOFs, including porous structure, tunable topology, high surface area, mechanical, thermal, and chemical stability, and containing transition metals endow them privilege as Hetero-Fenton catalysts [14,15]. Iron-based MOFs like MIL-88A revealed promising potential in Fenton degradation reactions owing to the nontoxicity and accessibility of iron species and the abundantly distributed metal nodes (Fe-O clusters) [16]. Nevertheless, the catalytic efficacy of neat iron-based MOFs requires further investigations, where they merely comprise ferric species with weak Fenton activity [17].

MXene is a newborn in the 2D-material family and has lately become a spotlight due to its individual features, comprising hydrophilicity, large surface area, chemical tunability, and good flexibility [18]. Hence, MXene has shown superior performance in the wastewater remediation sector, even by adsorption or catalysis [19]. Importantly, MXene is a biocompatible substance; therefore, it would not result in a secondary pollutant after the purification process [20]. MXene is prepared mainly by HF-etching of MAX; M symbols containing transition metal (Ti, Nb, and V), A represents group element (Sn, Al, and Si), and X could be carbon or nitrogen [21]. Nonetheless, the utilization of MXene as a Hetero-Fenton catalyst is still limited, and the catalytic mechanism is not perspicuous.

Tin ferrite (SnFe_2_O_4_) is one of the bright magnetic nanoparticles that possess propitious merits, such as its ferromagnetic nature, excellent mechanical and chemical stability, and good catalytic activity [22]. These merits make SnFe_2_O_4_ NPs an eminent Hetero-Fenton catalyst due to the collaboration between Sn and Fe to activate H_2_O_2_ and generate •OH radicals [23]. Furthermore, the excellent magnetic character provides a perfect catalyst separation after the degradation reaction by a magnet without the conventional separation ways that consume a longer time [24].

Accordingly, we intended to exploit the promising application prospects of MIL-88A, MXene, and SnFe_2_O_4_ in wastewater purification to fabricate the novel MIL-88A/SnFe_2_O_4_@MXene Hetero-Fenton catalyst. Characterization study of the morphological, chemical, structural, and physical characteristics of the MIL-88A/SnFe_2_O_4_@MXene composite was performed by XPS, SEM, XRD, FTIR, VSM, and Zeta potential. A series of Fenton-like degradation experiments of CR by the MIL-88A/SnFe_2_O_4_@MXene/H_2_O_2_ system proceeded to determine the optimal degradation conditions. Furthermore, the quenching test was carried out to indicate whether the degradation pathway is radical or nonradical. The degradation mechanism was presumed based on XPS analysis. In addition, the degradation pathway and the obtained intermediates were identified by GC–MS. Finally, the recyclability test was executed for five cycles to assess the catalyst’s durability.

## 2. Materials and Methods

### 2.1. Materials

The used chemicals in the preparation of MIL-88A/SnFe_2_O_4_@MXene and the characterization apparatus were summarized in Texts S1 and S2.

### 2.2. Synthesis of MIL-88A

MIL-88A was fabricated using a hydrothermal approach. Then, 2.5 g of FeCl_3_.6H_2_O and 1.1 g of FA were soaked in 60 mL of distilled water under potent stirring for 2 h. The Fe^3+^/FA solution was spilled into an autoclave and put in an oven at 85 °C for 24 h. The obtained orange powder was collected, washed with distilled water and ethanol, and dried at 70 °C [25].

### 2.3. Synthesis of MXene

MXene nanosheets were fabricated by the typical approach of the HF-etching of MAX. An amount of 5 g of MAX was slowly dipped into 150 mL of HF in a polytetrafluoroethylene container. Next, the MAX/HF solution was kept for 24 h to complete the reaction, then the obtained MXene was collected, washed with double distilled water, and dried at 70 °C for 12 h in a vacuum oven [26].

### 2.4. Synthesis of SnFe_2_O_4_ NPs

SnFe_2_O_4_ was fabricated via the simple co-precipitation approach. Dissolve 1.0 M of SnCl_2_ and 2.0 M of FeCl_3_.6H_2_O in 60 mL of distilled water under stirring for 30 min. An aqueous solution of NaOH was dropped over the Sn/Fe solution until the pH was 10, and then the temperature was elevated to 80 °C. After 2 h, the dark brown suspension was added to the autoclave and heated to 120 °C for 20 h. Ultimately, the SnFe_2_O_4_ particles were collected, washed until neutralization, and dried at 70 °C [27].

### 2.5. Synthesis of MIL-88A/SnFe_2_O_4_@MXene

**Scheme 1:**
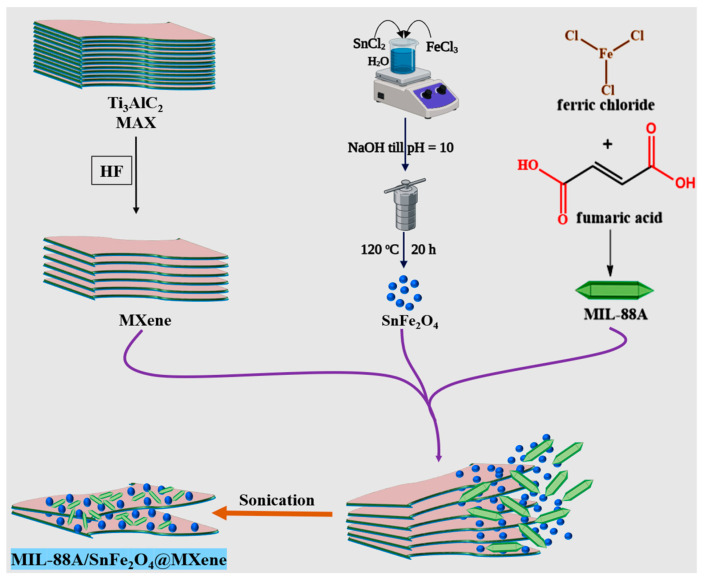
The fabrication procedure of the MIL-88A/SnFe_2_O_4_@MXene catalyst.

The MIL-88A/SnFe_2_O_4_@MXene catalyst was fabricated using the simple post-synthetic approach, as represented in Scheme 1. MIL-88A and SnFe_2_O_4_ NPs were dispersed in 20 mL of distilled water under sonication for 30 min. Then, MXene was mixed with the homogeneous suspension of MIL-88A/SnFe_2_O_4_ with continuous sonication for another 30 min. Finally, MIL-88A/SnFe_2_O_4_@MXene was collected and dried at 50 °C for 5 h. MIL-88A/SnFe_2_O_4_@MXene catalysts were prepared by different MIL-88A:MXene ratios: 1:3, 1:1, and 3:1.

### 2.6. Fenton-like Experiments

The optimization of the CR Fenton-like degradation process in the MIL-88A/SnFe_2_O_4_@MXene/H_2_O_2_ system was scrutinized as follows. (1) A comparison test was performed between MIL-88A, SnFe_2_O_4_, MXene, and MIL-88A/SnFe_2_O_4_@MXene to indicate the synergetic effect between the catalytic activities of MIL-88A, SnFe_2_O_4_, and MXene. (2) The pH impact on the catalytic degradation of CR was assessed in a pH range of 3–11. (3) The catalyst dose effect in the efficacy of the catalytic degradation reaction of CR molecules was studied at a dose ranging from 5 to 20 mg. (4) The system temperature influence on the CR degradation% was investigated by verifying the system temperature at the range of 20–50 °C. (5) The oxidant impact on the degradation aptitude of CR molecules was inspected at H_2_O_2_ ranging from 10 to 200 mg/L. The CR concentration was increased from 50 to 200 mg/L to define the catalytic activity of MIL-88A/SnFe_2_O_4_@MXene in low and high concentrations of CR molecules. The undegraded concentration of CR was detected by spectrophotometer at ʎmax = 500 nm, and the degradation% was calculated from Equation (1).

(1) $$\mathrm{Degradation}\%=\frac{C_{o}-C_{t}}{C_{o}}\times 100$$

*C_o_* is the initial CR concentration, and *C_t_* is the undegraded concentration of CR after the Fenton-like degradation process.

### 2.7. Cycling Test

A cycling test was performed on the magnetic MIL-88A/SnFe_2_O_4_@MXene catalyst for five catalytic runs. After completing the catalytic run, the MIL-88A/SnFe_2_O_4_@MXene catalyst was collected and washed with acetone to remove the residual CR molecules and the degradation products. After drying the catalyst at 50 °C for 6 h, the regenerated catalyst was used in the next catalytic degradation cycle of CR molecules.

## 3. Results and Discussions

### 3.1. Characterization of MIL-88A/SnFe_2_O_4_@MXene

#### 3.1.1. FTIR

**Figure 1:**
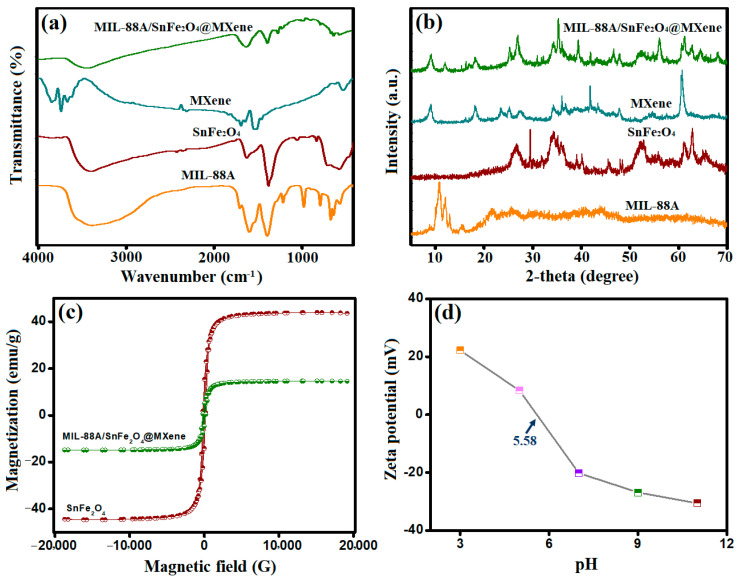
(**a**) FTIR, (**b**) XRD, (**c**) VSM, and (**d**) Zeta potential of the magnetic MIL-88A/SnFe_2_O_4_@MXene and its authentic components.

The active functional groups on the MIL-88A/SnFe_2_O_4_@MXene surface were investigated by the FTIR spectra (Figure 1a). The absorption bands of MIL-88A that were observed at 1605 and 1395 cm^−1^ are allocated to the asymmetric and symmetric COOH vibrational, sequentially. The belonging absorption band of the Fe-O bond appeared at 571 cm^−1^, which confirms the bond formation between the iron species and FA. The absorption bands at 1705 cm^−1^ are assigned to the C=O group; in addition, the related band to the O-H group manifested at 3375 cm^−1^. The carbon-containing groups of MIL-88A appeared at 982 cm^−1^ (C–H) and 1716 cm^−1^ (C–C). The spectrum of SnFe_2_O_4_ clarified its characteristic peaks at wavenumbers of 707 and 580 cm^−1^, which corresponded to Sn–O and Fe–O, respectively. The absorption band at 1630 cm^−1^ corresponds to O–H bending vibrational, and the broadband at 3395 cm^−1^ relates to O–H stretching. The spectrum of MXene illustrated the belonging absorption peaks of Ti–C, C–F, and Ti–O at the wavenumbers of 523, 1249, and 630 cm^−1^, respectively. The accompanying bands of O–H bending appeared at 1456 cm^−1^, and O–H stretching manifested at 3647 cm^−1^. The distinctive absorption bands of MIL-88A, SnFe_2_O_4_, and MXene were observed in the spectrum of MIL-88A/SnFe_2_O_4_@MXene, ensuring their binding to form the composite.

#### 3.1.2. XRD

Figure 1b illustrates the crystallite characters of MIL-88A, SnFe_2_O_4_, MXene, and MIL-88A/SnFe_2_O_4_@MXene. The crystallographic spectrum of MIL-88A revealed its characteristic peaks at 10.77° and 11.96° that accompany the planes of 100 and 101, respectively [17]. The crystallographic profile of SnFe_2_O_4_ shows its diffraction peaks at 26.61°, 29.47°, 33.78°, 52.47°, 61.13°, and 62.77°, which coincide with the planes of 111, 220, 331, 422, 511, and 440, respectively. The crystallographic spectrum of MXene, the XRD peaks at 9.03°, 18.3°, 25.4°, 34.3°, 41.8°, and 60.6°, corresponded to the planes of 002, 006, 008, 0010, 0012, and 110, sequentially [28]. The pattern of MIL-88A/SnFe_2_O_4_@MXene confirmed the binding of MIL-88A, SnFe_2_O_4_, and MXene, where the distinguishing peaks of them were observed in the composite pattern.

#### 3.1.3. VSM

The magnetism hysteresis loops of SnFe_2_O_4_ and MIL-88A/SnFe_2_O_4_@MXene are represented in Figure 1c. The coercivity magnitudes of SnFe_2_O_4_ and MIL-88A/SnFe_2_O_4_@MXene were 188.96 and 176.95 G, reflecting their soft ferromagnetic character. Moreover, the saturation magnetization values of SnFe_2_O_4_ and MIL-88A/SnFe_2_O_4_@MXene were 43.95 and 14.19 emu/g, respectively, clarifying a decline in the magnetic property of MIL-88A/SnFe_2_O_4_@MXene compared to SnFe_2_O_4_. This observation owes to the nonmagnetic properties of MIL-88A and MXene. Despite the lower magnetism of MIL-88A/SnFe_2_O_4_@MXene than the authentic SnFe_2_O_4_, the magnetism of the composite enables it to be an easily separable catalyst.

#### 3.1.4. Zeta Potential

The carried charges on the surface of the MIL-88A/SnFe_2_O_4_@MXene composite were detected using zeta potential analysis, as represented in Figure 1d. The zeta potential results depicted that MIL-88A/SnFe_2_O_4_@MXene carried zero charge when pH = 5.58. This finding implied that MIL-88A/SnFe_2_O_4_@MXene carried positive charges when the pH medium was lower than 5.58 and attained the maximal positive charges (22.21 mV) at pH = 3. When pH was elevated over 5.58, the net charges on the composite’s surface were negative, reaching −30.60 mV at pH = 11.

#### 3.1.5. SEM

**Figure 2:**
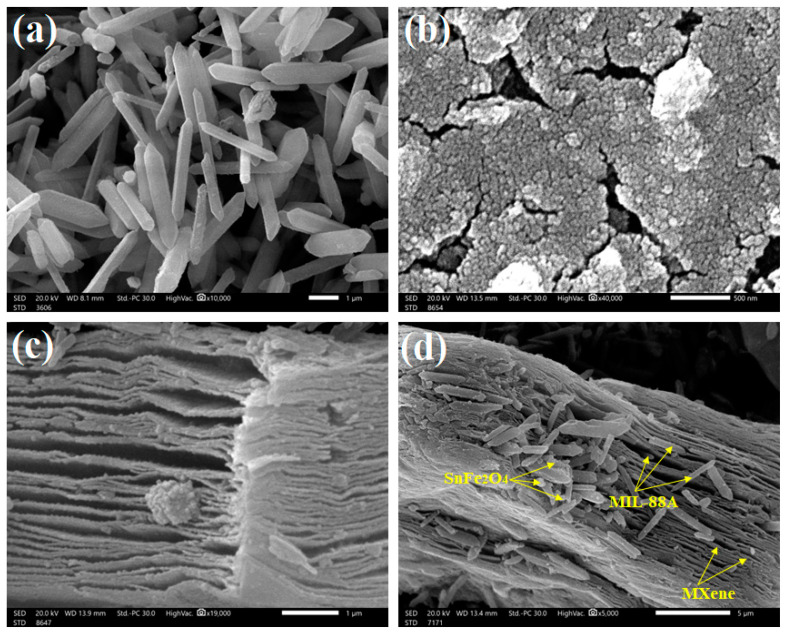
SEM images of (**a**) MIL-88A, (**b**) SnFe_2_O_4_, (**c**) MXene, and (**d**) MIL-88A/SnFe_2_O_4_@MXene composite.

The outer shapes of MIL-88A, SnFe_2_O_4_, MXene, and MIL-88A/SnFe_2_O_4_@MXene were studied using SEM (Figure 2a–d). It was observed that MIL-88A has a rod-like shape, which is the typical shape of the hydrothermally fabricated MIL-88A. The particles of the SnFe_2_O_4_ shape are spheroidal, forming high aggregations owing to the high ferromagnetic character of SnFe_2_O_4_, as proven by VSM. The SEM of MXene elucidates exfoliated thin sheets with a rough surface, endowing it a feature to be a good supporter. The SEM of MIL-88A/SnFe_2_O_4_@MXene shows the distribution of the rod-like shape of MIL-88A and the spheroidal SnFe_2_O_4_ on the sheets of MXene.

#### 3.1.6. XPS

**Figure 3:**
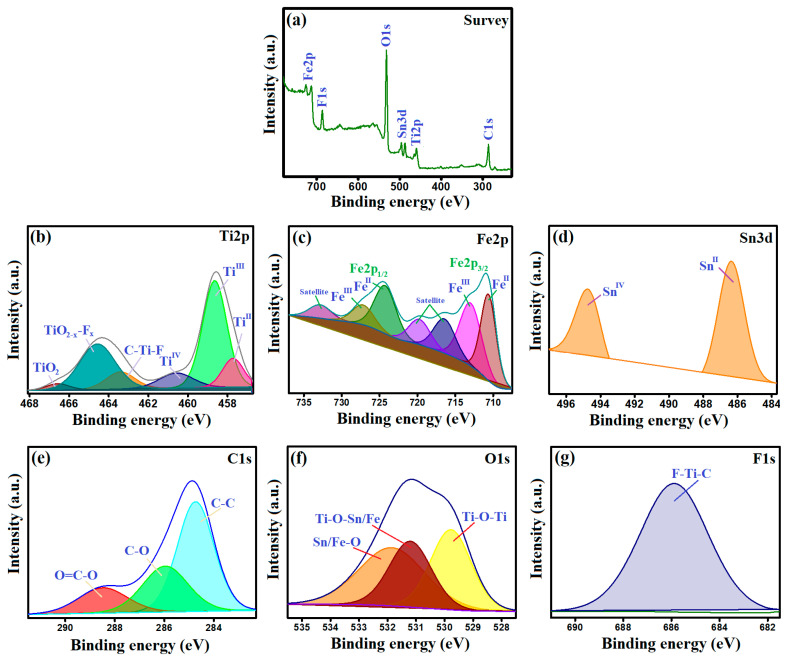
XPS analysis of MIL-88A/SnFe_2_O_4_@MXene; (**a**) Survey, (**b**) Ti2p, (**c**) Fe2p, (**d**) Sn3d, (**e**) C1s, (**f**) O1s, and (**g**) F1s.

The XPS survey of MIL-88A/SnFe_2_O_4_@MXene depicts that it is composed of carbon, iron, tin, titanium, oxygen, and fluoride, where their belonging peaks appeared at 286.17, 712.45, 487.27, 459.23, 531.89, and 686.54 eV, respectively (Figure 3a). The titanium spectrum (Figure 3b) revealed the peaks of Ti^II^, Ti^III^, Ti^IV^, C–Ti–F, TiO_2-x_–F_x_, and TiO_2_ at 457.70, 458.63, 460.61, 463.35, 464.54, and 466.64 eV, respectively [29,30,31]. The iron spectrum revealed ferric peaks of Fe^III^2p_3/2_ and Fe^III^2p_1/2_ at 712.97 and 724.25 eV, respectively (Figure 3c). In addition, ferrous peaks manifested at 710.61 (Fe^II^2p_3/2_) and 727.17 eV (Fe^II^2p_1/2_) [32]. The tin spectrum (Figure 3d) revealed the characteristic peaks of Sn^II^ and Sn^IV^ at 486.37 and 494.64 eV, sequentially [33]. The carbon spectrum (Figure 3e) represents the carbon-based functional groups of C–O, O=C–O, and C–C at the binding energies of 285.96, 288.48, and 284.74 eV, respectively. The oxygen spectrum exhibited peaks at 531.85, 529.78, and 531.20 eV, which are assigned to Sn/Fe–O, Ti–O–Ti, and Ti–O–Sn/Fe, sequentially (Figure 3f). The fluoride spectrum (Figure 3g) shows a peak at 685.88 eV, which coincides with F–Ti–C.

### 3.2. Optimization of the CR Fenton-like Degradation Process

#### 3.2.1. Comparison Test

**Figure 4:**
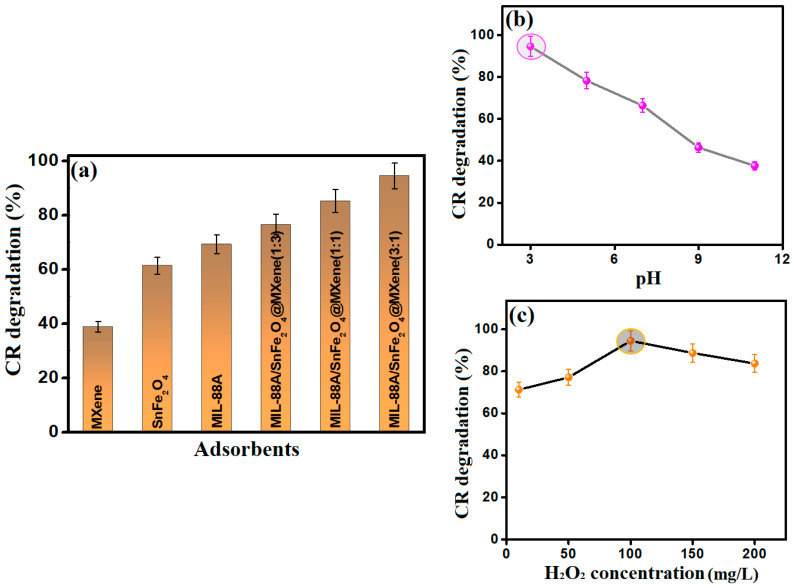
(**a**) The catalytic degradation of CR using various catalysts (CR concentration = 50 mg/L, catalyst mass = 10 mg, pH = 3, H_2_O_2_ concentration = 100 mg/L, t = 30 min, and temperature = 20 °C), (**b**) Effect of the pH medium (CR concentration = 50 mg/L, catalyst mass = 10 mg, pH = 3–11, H_2_O_2_ concentration = 100 mg/L, t = 30 min, and temperature = 20 °C), and (**c**) Effect of H_2_O_2_ concentration (CR concentration = 50 mg/L, catalyst mass = 10 mg, pH = 3, H_2_O_2_ concentration = 10–200 mg/L, t = 30 min, and temperature = 20 °C).

The Fenton-like degradation reaction of CR molecules was studied using varied catalysts: MIL-88A, SnFe_2_O_4_, MXene, and MIL-88A/SnFe_2_O_4_@MXene as the same catalytic reaction conditions (Figure 4a). The experimental work denoted that the Fenton-like degradation efficacy of CR molecules by MIL-88A, SnFe_2_O_4_, and MXene were 69.30, 61.39, and 38.85% after 30 min, respectively. Surprisingly, a drastic enhancement was noticed in the catalytic activity of MIL-88A/SnFe_2_O_4_@MXene composites towards the degradation of CR, which were 76.55, 85.25, and 94.54% when the MIL-88A: MXene ratios were 1:3, 1:1, and 3:1, sequentially. These results reflected the synergistic effect between the catalytic activities of MIL-88A, SnFe_2_O_4_, and MXene. The MIL-88A/SnFe_2_O_4_@MXene composite with the MIL-88A: MXene ratio = 3:1 was selected to complete the rest of the CR Fenton-like degradation experiments.

#### 3.2.2. The Impact of the Catalytic pH Medium

The pH of the catalytic medium significantly impacts the Fenton-like degradation aptitude of CR dye; therefore, the degradation process was scrutinized in a wide pH scale (3–11) for 30 min, as demonstrated in Figure 4b. It was deduced that a highly acidic medium is the best pH medium to degrade CR since its degradation% was 94.54% after 30 min at pH = 3. Whereas, when pH is augmented to near alkaline media, hydroxy iron may precipitate, as elucidated in Equation (2), declining the concentration of the iron species in the catalytic medium. Furthermore, when pH increased to alkaline media, the Fenton-like degradation of CR dwindled, which is most likely due to the interaction between the abundant hydroxyl ions with H_2_O_2_ to form hydroperoxy anions (Equations (3) and (4)), which possess a superior affinity to metal species. This is in addition to the possible auto-decomposition of hydrogen peroxide to carbon dioxide and water at high alkaline media (Equation (5)), which decreases the produced amounts of •OH radicals.

Fe^2+^ + •OH → Fe^3+^ + OH^−^ → Fe(OH)_3_ (2)

H_2_O_2_ + OH^−^ → OOH^−^ + H_2_O (3)

M + OOH^−^ → M…^•^OOH (4)

2H_2_O_2_ → O_2_ + 2H_2_O (5)

#### 3.2.3. The Impact of the H_2_O_2_ Concentration

Indeed, H_2_O_2_ is the dynamic force of the Fenton/Fenton-like reactions, so the influence of increasing the H_2_O_2_ concentrations on the degradation% of CR dye was studied, as shown in Figure 4c. An amelioration was observed in the degradation% of CR molecules from 71.25 to 94.54% after 30 min when the concentration of H_2_O_2_ elevated from 10 to 100 mg/L, which could be explained by fostering the production of •OH radicals with the augmentation of the oxidant concentrations. Nevertheless, over-raising in the H_2_O_2_ concentrations beyond 100 mg/L worsened the Fenton-like degradation aptitude of CR, which could be due to the possibility of interacting the excess concentrations of H_2_O_2_ with the presented •OH to form hydroperoxy radicals (Equation (6)). This hydroperoxy could attack the distributed •OH in the catalytic medium and degrade to water and oxygen, as elucidated in Equation (7).

H_2_O_2_ + ^•^OH → HOO^•^ + H_2_O (6)

HOO^•^ + ^•^OH → H_2_O + O_2_ (7)

#### 3.2.4. The Impact of MIL-88A/SnFe_2_O_4_@MXene Dose

**Figure 5:**
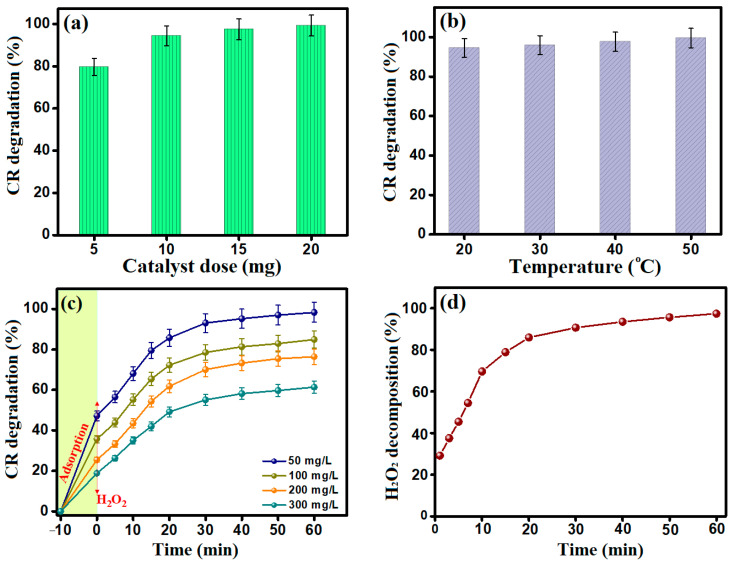
(**a**) Effect of catalyst dose (CR concentration = 50 mg/L, catalyst mass = 5–20 mg, pH = 3, H_2_O_2_ concentration = 100 mg/L, t = 30 min, and temperature = 20 °C), (**b**) Effect of the reaction temperature (CR concentration = 50 mg/L, catalyst mass = 10 mg, pH = 3, H_2_O_2_ concentration = 100 mg/L, t = 30 min, and temperature = 20–50 °C), (**c**) Effect of the CR concentrations (CR concentration = 50–300 mg/L, catalyst mass = 10 mg, pH = 3, H_2_O_2_ concentration = 100 mg/L, t = 70 min, and temperature = 20 °C), and (**d**) H_2_O_2_ decomposition (CR concentration = 50 mg/L, catalyst mass = 10 mg, pH = 3, H_2_O_2_ concentration = 100 mg/L, t = 60 min, and temperature = 20 °C).

Figure 5a reveals the impact of the catalyst dosage study on the efficacy of the Fenton-like degradation reaction of CR dye. A noticeable improvement in the degradation% of CR from 79.90 to 99.35% was recorded after 30 min when raising the MIL-88A/SnFe_2_O_4_@MXene dose. This catalytic activity enhancement with elevating the catalyst dose is attributed to boosting the generation of the •OH concentration, which directly reinforced the degradation of CR molecules.

#### 3.2.5. The Impact of the Temperature of the Catalytic System

The influence of the temperature of the catalytic system on the degradation of CR dye by MIL-88A/SnFe_2_O_4_@MXene catalyst was scrutinized at a temperature range of 20–50 °C (Figure 5b) at pH 3 and 30 min of reaction time. The Fenton-like degradation of CR increased until almost 100% when the temperature of the catalytic system elevated to 50 °C. This performance could be ascribed to boosting the interaction between MIL-88A/SnFe_2_O_4_@MXene and H_2_O_2_ at higher temperatures, giving rise to fostering the production of the •OH radicals [34].

#### 3.2.6. The Impact of the Concentration of CR Molecules

The degradation activity of MIL-88A/SnFe_2_O_4_@MXene towards different CR concentrations was studied within 1 h (Figure 5c). A synergistic effect was noticed between adsorption and Fenton-like degradation processes; the first step involved the adsorption of CR dye onto the surface of the MIL-88A/SnFe_2_O_4_@MXene catalyst in which the adsorption% values of CR were 47.25, 35.73, 25.54, and 18.90% after 10 min when the CR concentrations were 50, 100, 200, and 300 mg/L, respectively. Then, the second step began after adding H_2_O_2_, revealing that the Fenton-like degradation% of CR dye attained 98.31, 84.94, 76.43, and 61.37% at CR concentrations of 50, 100, 200, and 300 mg/L, respectively, after 60 min.

#### 3.2.7. Kinetic Study of H_2_O_2_ Decomposition

The decomposition of H_2_O_2_ by MIL-88A/SnFe_2_O_4_@MXene catalyst controls the concentration of the generated •OH radicals and directly the efficacy of the degradation of CR molecules. Consequently, the kinetics of the H_2_O_2_ decomposition by MIL-88A/SnFe_2_O_4_@MXene was scrutinized, in the absence of CR dye (Figure 5d). The decomposition percent of H_2_O_2_ with a concentration of 100 mg/L reached 97.04% after 60 min.

### 3.3. Kinetic Study

#### 3.3.1. CR Decomposition

The experimental results from the Fenton-like degradation of CR in the catalytic MIL-88A/SnFe_2_O_4_@MXene/H_2_O_2_ system were analyzed using First-order and Second-order models (Equations (8) and (9)).

(8) $$\ln\frac{C_{t}}{C_{o}}=-k_{1}t$$

(9) $$\frac{1}{C_{t}}=\frac{1}{C_{o}}+k_{2}t$$

*C_t_* and *C_o_* are the concentrations of CR dye at a time *t* and initial reaction time, respectively. *k*_1_ and *k*_2_ are the First-order and Second-order rate constants, sequentially.

**Figure 6:**
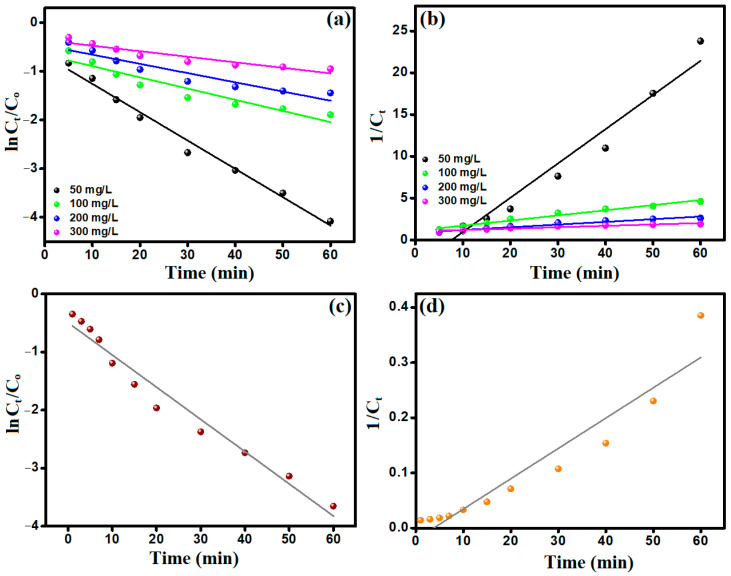
(**a**) First-order, (**b**) Second-order of the Fenton-like degradation of CR, (**c**) First-order and (**d**) Second-order of H_2_O_2_ decomposition by MIL-88A/SnFe_2_O_4_@MXene catalyst.

The kinetic analysis (Figure 6a,b) implied that obeying the Fenton-like degradation of CR by MIL-88A/SnFe_2_O_4_@MXene catalyst to the Second-order model owes to the higher R^2^ values that are derived from the Second-order model (Table 1) [35]. Moreover, the Fenton-like rate constants at the CR concentrations of 50, 100, 200, and 300 mg/L were 0.4098, 0.0615, 0.0322, and 0.0166 min^−1^, respectively, reflecting the high reaction rate constant, at low CR concentration.

#### 3.3.2. H_2_O_2_ Decomposition

The decomposition of H_2_O_2_ by MIL-88A/SnFe_2_O_4_@MXene catalyst was inspected by First-order and Second-order models. Figure 6c,d imply that the decomposition of H_2_O_2_ obeyed the First-order model, in which the obtained R^2^ values from First-order and Second-order plots were 0.965 and 0.904, respectively. Furthermore, the rate constant of H_2_O_2_, based on the First-order model, was 0.0555 min^−1^.

### 3.4. Scavenging Test

**Figure 7:**
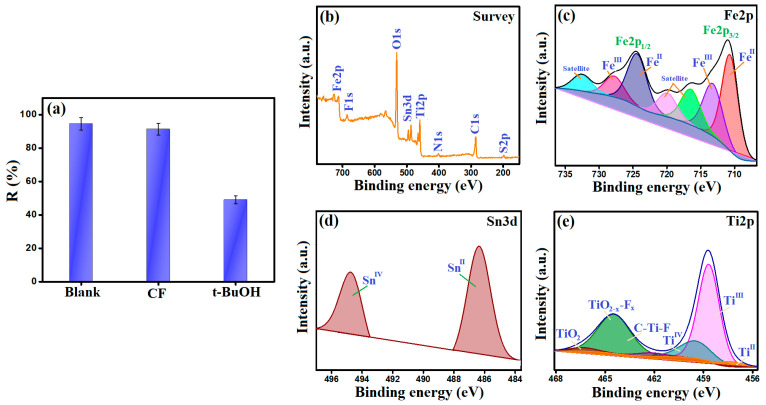
(**a**) Scavenging test, (**b**) XPS survey of the used MIL-88A/SnFe_2_O_4_@MXene, (**c**) Fe2P spectrum, (**d**) Sn3d spectrum, and (**e**) Ti2p spectrum.

Figure 7a illustrates the scavenging test of the Fenton-like degradation reaction of CR by MIL-88A/SnFe_2_O_4_@MXene catalyst. Generally, chloroform (CF) quenches O_2_•^−^ radicals, and t-butanol (t-BuOH) hinders •OH radicals [36]. The scavenging test revealed a trivial effect of CF on the efficacy of the Fenton-like reaction of CR by the MIL-88A/SnFe_2_O_4_@MXene/H_2_O_2_ system. On the contrary, t-BuOH dwindled almost half of the activity of MIL-88A/SnFe_2_O_4_@MXene towards the degradation of CR molecules. These results proved that •OH radicals are the controlling reactive groups on the Fenton-like reaction of CR molecules inside the MIL-88A/SnFe_2_O_4_@MXene/H_2_O_2_ system. Additionally, the radical pathway is the following pathway of the Fenton-like degradation reaction of CR, not the oxygen vacancies pathway [37].

### 3.5. Degradation Mechanism

In the light of the experimental work, the degradation of CR molecules by MIL-88A/SnFe_2_O_4_@MXene composite proceeded by the synergistic effect between adsorption and Fenton-like processes. Firstly, the adsorption of CR molecules onto the active binding sites of MIL-88A/SnFe_2_O_4_@MXene was proved from the XPS survey of the used composite (Figure 7b), where the belonging peaks of sulfur- and nitrogen-containing CR appeared at the binding energy of 169.95 and 401.11 eV, respectively. The adsorption process of CR onto MIL-88A/SnFe_2_O_4_@MXene was supposed to occur via the coordination bonds between the amine-containing CR molecules and the metal species of MIL-88A/SnFe_2_O_4_@MXene: Ti, Sn, and Fe. Furthermore, the potent electrostatic interaction could occur between the cationic active sites of MIL-88A/SnFe_2_O_4_@MXene (zeta potential = 22.21 mV at pH = 3) and the anionic CR molecules. Notably, the H-bonding drastically contributed to the adsorption of CR onto MIL-88A/SnFe_2_O_4_@MXene since the oxygen and nitrogen atoms of CR molecules and the abundant hydrogen atoms onto the composite surface could attach via H-bonds. In addition, the oxygenated functional groups onto the backbone of MIL-88A/SnFe_2_O_4_@MXene could form H-bonds with the hydrogen atoms of the CR molecules.

Secondly, the Fenton-like degradation process of CR molecules proceeded by the activation of H_2_O_2_ as follows.

(i) Fe^II^ species could transfer electrons to activate H_2_O_2_ and form •OH radicals, following the Haber–Weiss mechanism, as represented in Equation (10). This proposition was proved by the blue shift of the Fe2p spectrum of the used MIL-88A/SnFe_2_O_4_@MXene catalyst (Figure 7c). In addition, the Fe^III^/Fe^II^ ratio of the used catalyst was found to be 0.549, while the ratio of the pure catalyst was 0.264, reflecting the increase in the Fe^III^ amounts after the Fenton-like degradation of CR and contributing of Fe^II^ species in the H_2_O_2_ activation.

Fe^II^ + H_2_O_2_ → Fe^III^ + •OH + ^−^OH            (E^o^ Fe^II^_/_Fe^III^ = 0.77 V) (10)

(ii) The Sn^II^ ions play a dual role in the Fenton-like degradation of CR molecules since they could provide the needed electrons to activate H_2_O_2_ and produce •OH radicals (Equation (11)). The participation of Sn^II^ species in the H_2_O_2_ activation was confirmed by the detected shifting in the Sn3d peaks and the increase in the Sn^IV^/Sn^II^ ratio after the Fenton-like reaction from 0.599 to 0.674 (Figure 7d); additionally, the ability of Sn^II^ to recover Fe^III^ to Fe^II^, where the redox potential (E^o^) of Sn^II^/Sn^IV^ is 0.14 V, and the E^o^ of Fe^II^/Fe^III^ is 0.77 V (Equation (12)).

Sn^II^ + 2 H_2_O_2_ → Sn^IV^ + 2 •OH + 2 ^−^OH            (E^o^ Sn^II^_/_Sn^IV^ = 0.14V) (11)

**Figure 8:**
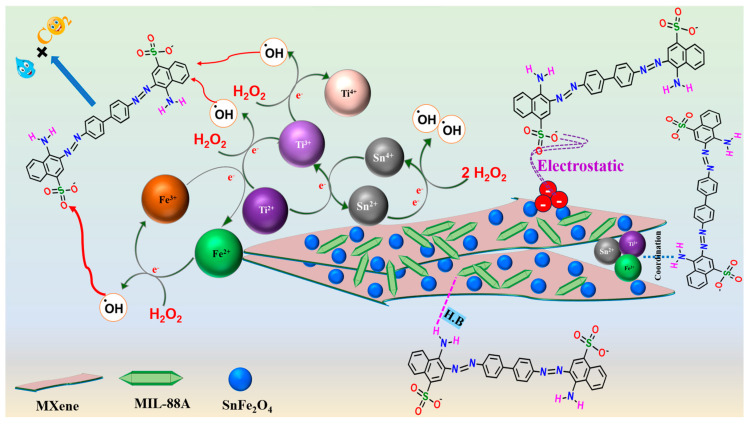
The adsorption and Fenton-like degradation of CR by MIL-88A/SnFe_2_O_4_@MXene.

Sn^II^ + 2Fe^III^ → 2Fe^II^ + Sn^IV^ (12)

(iii) The abundant Ti species (Figure 7e) also contribute to the •OH generation and recover both Fe^II^ and Sn^II^ species. The Ti^II^ and Ti^III^ ions activated H_2_O_2_, as elucidated in Equations (13) and (14). The lower E^o^ value of Ti^II^/Ti^III^ compared to Ti^III^/Ti^IV^ implies a higher affinity of Ti^II^ to activate H_2_O_2_ than Ti^III^. Also, the Ti^III^/Ti^II^ ratio drastically increased from 1.3 to 16.87, while the ratio of Ti^III^/Ti^IV^ trivially augmented from 0.274 to 0.277, which reflected the lower contribution of Ti^III^ in the Fenton-like degradation of CR. Such a low E^o^ value of Ti^II^/Ti^III^ enables it to recover Fe^II^ and Sn^II^ species and sustains the Fenton-like reaction for a long time (Equations (15) and (16)). These suggestions explained the dramatic decline in the atomic percent of Ti^II^ ions and denoted the significance of MXene in the reducibility of iron and tin [38]. Figure 8 exhibits the adsorption and Fenton-like degradation mechanisms of CR by MIL-88A/SnFe_2_O_4_@MXene.

Ti^II^ + H_2_O_2_ → Ti^III^ + •OH + ^−^OH            (E^o^ Ti^II^_/_Ti^III^ = 0.10 V) (13)

Ti^III^ + H_2_O_2_ → Ti^IV^ + •OH + ^−^OH           (E^o^ Ti^III^_/_Ti^IV^ = 1.2 V) (14)

Ti^II^ + Fe^III^ → Ti^III^ + Fe^II^ (15)

2 Ti^II^ + Sn^IV^ → 2Ti^III^ + Sn^II^ (16)

### 3.6. Degradation Pathway of CR Molecules

**Figure 9:**
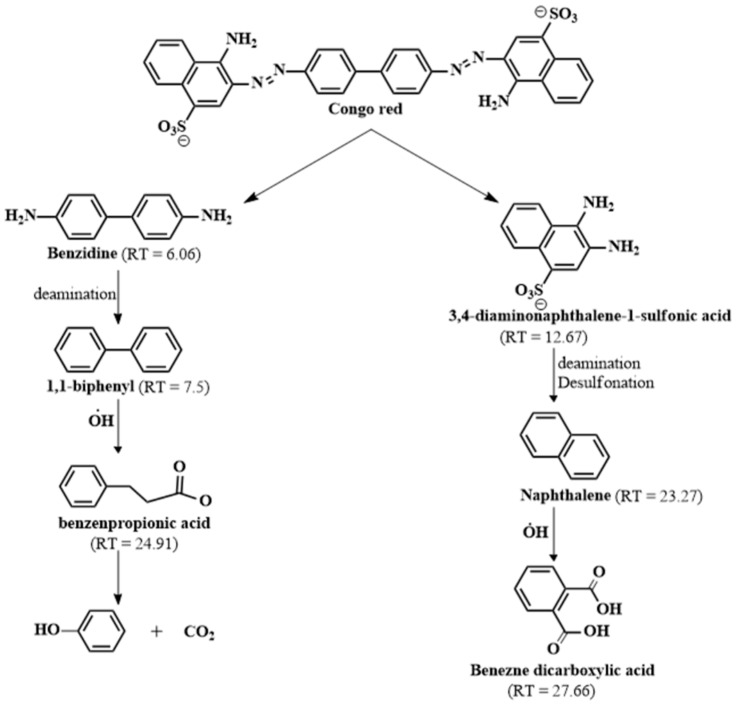
Proposed degradation mechanism of CR molecules based on GC–MS analysis.

The degradation pathway of the Fenton-like degradation of CR molecules by MIL-88A/SnFe_2_O_4_@MXene was detected by GC–MS (Figure S1). The decomposition of the CR molecules was supposed to proceed by cleaving of the azo bond, as elucidated in Figure 9, forming benzidine in one pathway at a retention time (RT) of 6.06 min, and 3,4-diaminonaphthalene-1-sulfonic acid in another pathway at RT = 12.67 min. In the first pathway, benzidine converted to 1,1′-biphenyl (RT = 7.5 min) via a deamination reaction, and then it further oxidized to benzene propanoic acid (RT = 24.91 min) and phenol (RT = 6.98 min). In the second pathway, 3,4-diamino naphthalene-1-sulfonic acid converted to naphthalene (RT = 23.27 min) via desulfonation and deamination reactions that oxidized to benzene dicarboxylic acid (RT = 27.66 min).

### 3.7. Recyclability Test

**Figure 10:**
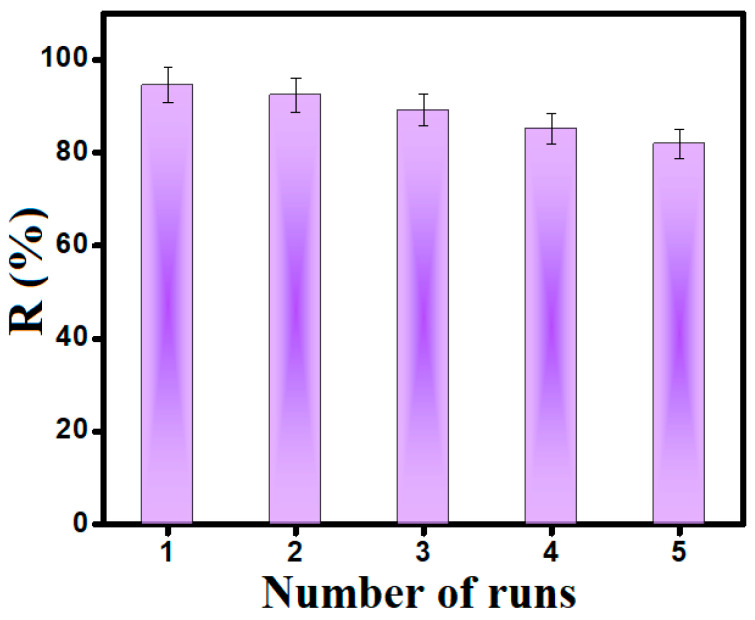
Recyclability test of MIL-88A/SnFe_2_O_4_@MXene during five runs of the Fenton-like degradation reaction of CR molecules.

The recyclability test was performed on MIL-88A/SnFe_2_O_4_@MXene after the Fenton-like degradation reaction of CR molecules for five runs, as represented in Figure 10. The experimental result indicated the pre-eminent recyclability of the MIL-88A/SnFe_2_O_4_@MXene catalyst, in which its catalytic activity toward the Fenton-like degradation of CR dwindled by 12.61% after the 5th catalytic run. This finding may be ascribed to the magnetic property of the catalyst that eases its separation without weight loss. Furthermore, the continuous redox cycle between Fe/Sn/Ti species in the MIL-88A/SnFe_2_O_4_@MXene catalyst increases its activity for a longer time.

### 3.8. Leaching Test

For confirming the durability of the MIL-88A/SnFe_2_O_4_@MXene catalyst, the metals leaching from the catalyst were determined by ICP-OES. It was recorded that the leaching concentrations of Fe, Sn, and Ti were 0.01, 0.007, and 0.009 mg/L, respectively. These results indicated the stability of the chemical structure of the MIL-88A/SnFe_2_O_4_@MXene catalyst. Furthermore, these low leaching concentrations of the metal-containing MIL-88A/SnFe_2_O_4_@MXene reflected the nontoxicity of the catalyst, where it does not result in the formation of secondary pollutants.

## 4. Conclusions

The MIL-88A/SnFe_2_O_4_@MXene catalyst revealed eminent Fenton-like degradation performance toward CR molecules, where the degradation% almost attained 100% during 60 min. Furthermore, the experimental work exhibited the synergistic effect between adsorption and Fenton-like processes to degrade CR molecules since the adsorption% was 47.25% at 50 mg/L of the CR. The superior Fenton-like degradation of CR was recorded at the catalytic reaction conditions of pH = 3, catalyst dose = 10 mg, CR concentration = 50 mg/L, H_2_O_2_ concentration = 100 mg/L, and system temperature = 55 °C. The kinetic study denoted fitting the CR decomposition to the Second-order model and H_2_O_2_ decomposition to the First-order model. The scavenger test implied the predominance of •OH radicals on the CR degradation by the MIL-88A/SnFe_2_O_4_@MXene/H_2_O_2_ catalytic system. The adsorption mechanism of the CR degradation was presumed to proceed by two processes: the adsorption process that occurred via H-bonding, coordination bond, and electrostatic interaction between CR molecules and MIL-88A/SnFe_2_O_4_@MXene, in addition to the Fenton-like degradation that is controlled by the formation of the sustainable redox cycle between Sn, Ti, and Fe species. Interestingly, MIL-88A/SnFe_2_O_4_@MXene showed an excellent Fenton-like degradation behavior toward the CR molecules during five sequential runs, reflecting the superior recyclability of the catalyst. Nevertheless, the high cost of MXene and MIL-88A limits the study to integration from a lab scale. Consequently, excessive investigations are still needed to find preparation approaches to MXene and MOFs with a lower capital cost.

## Figures and Tables

**Table 1:** Derived parameter of the kinetic study of the Fenton-like degradation of CR by MIL-88A/SnFe_2_O_4_@MXene catalyst.

Kinetic Model	CR Concentrations (mg/L)			
	50	100	200	300
**First-order**				
k_1_	0.0582	0.0229	0.0189	0.0114
R^2^	0.985	0.894	0.882	0.870
**Second-order**				
k_2_	0.4098	0.0615	0.0322	0.0166
R^2^	0.949	0.974	0.946	0.918

## Data Availability

Data will be available upon request.

## Supplementary Materials

The following supporting information can be downloaded at: https://www.mdpi.com/article/10.3390/nano14010054/s1, Text S1. The used chemicals in the preparation of MIL-88A/SnFe_2_O_4_@MXene composite; Text S2. Characterization tools; Figure S1. GC-MS of CR molecules after the Fenton-like degradation by MIL-88A/SnFe_2_O_4_@MXene.

## References

[1] Al-Tohamy R., Ali S.S., Li F., Okasha K.M., Mahmoud Y.A.-G., Elsamahy T., Jiao H., Fu Y., Sun J. (2022). A critical review on the treatment of dye-containing wastewater: Ecotoxicological and health concerns of textile dyes and possible remediation approaches for environmental safety. Ecotoxicol. Environ. Saf..

[2] El-Monaem E.M.A., Ayoup M.S., Omer A.M., Hammad E.N., Eltaweil A.S. (2023). Sandwich-like construction of a new aminated chitosan Schiff base for efficient removal of Congo red. Appl. Water Sci..

[3] Shindhal T., Rakholiya P., Varjani S., Pandey A., Ngo H.H., Guo W., Ng H.Y., Taherzadeh M.J. (2021). A critical review on advances in the practices and perspectives for the treatment of dye industry wastewater. Bioengineered.

[4] Zhou H., Qiu Y., Yang C., Zang J., Song Z., Yang T., Li J., Fan Y., Dang F., Wang W. (2022). Efficient Degradation of Congo Red in Water by UV-Vis Driven CoMoO_4_/PDS Photo-Fenton System. Molecules.

[5] Shen Y., Zhou Y., Zhang Z., Xiao K. (2017). Cobalt–copper oxalate nanofibers mediated Fenton degradation of Congo red in aqueous solutions. J. Ind. Eng. Chem..

[6] Solano A.M.S., Garcia-Segura S., Martínez-Huitle C.A., Brillas E. (2015). Degradation of acidic aqueous solutions of the diazo dye Congo Red by photo-assisted electrochemical processes based on Fenton’s reaction chemistry. Appl. Catal. B Environ..

[7] Ismail G.A., Sakai H. (2022). Review on effect of different type of dyes on advanced oxidation processes (AOPs) for textile color removal. Chemosphere.

[8] Alharbi R.A., Alminderej F.M., Al-Harby N.F., Elmehbad N.Y., Mohamed N.A. (2023). Preparation and characterization of a new bis-uracil chitosan-based hydrogel as efficient adsorbent for removal of anionic Congo red dye. Polymers.

[9] Modwi A., Mustafa B., Toghan A., Taha K.K. (2023). Scalable fabrication and characterization of Y_2_O_3_@g-C_3_N_4_ nanocomposite for the enhancement of photocatalytic removal of Congo red dye under visible light. J. Mater. Sci. Mater. Electron..

[10] Al-Harby N.F., Albahly E.F., Mohamed N.A. (2022). Synthesis and characterization of novel uracil-modified chitosan as a promising adsorbent for efficient removal of Congo red dye. Polymers.

[11] Nidheesh P.V., Ganiyu S.O., Martínez-Huitle C.A., Mousset E., Olvera-Vargas H., Trellu C., Zhou M., Oturan M.A. (2023). Recent advances in electro-Fenton process and its emerging applications. Crit. Rev. Environ. Sci. Technol..

[12] Cheng M., Song W., Ma W., Chen C., Zhao J., Lin J., Zhu H. (2008). Catalytic activity of iron species in layered clays for photodegradation of organic dyes under visible irradiation. Appl. Catal. B Environ..

[13] Liao J., Li H., Zhang X., Xiao D., Qiang N. (2015). Facile fabrication of Ti supported CuO film composed of bamboo-leaf-like nanosheets and their high catalytic performance in the oxidative degradation of methylene blue with hydrogen peroxide. Appl. Catal. A Gen..

[14] Cui W., Kang X., Zhang X., Cui X. (2019). Gel-like ZnO/Zr-MOF (bpy) nanocomposite for highly efficient adsorption of Rhodamine B dye from aqueous solution. J. Phys. Chem. Solids.

[15] He J., Zhang Y., Zhang X., Huang Y. (2018). Highly efficient Fenton and enzyme-mimetic activities of NH2-MIL-88B (Fe) metal organic framework for methylene blue degradation. Sci. Rep..

[16] Wang H., Yu S., Meng X., Wang Z., Gao T., Xiao S. (2022). Facile synthesis of fumarate-type iron-cobalt bimetallic MOFs and its application in photo-Fenton degradation of organic dyes. J. Solid State Chem..

[17] Ren G., Zhao K., Zhao L. (2020). A Fenton-like method using ZnO doped MIL-88A for degradation of methylene blue dyes. RSC Adv..

[18] Cui Y., Zhang D., Shen K., Nie S., Liu M., Huang H., Deng F., Zhou N., Zhang X., Wei Y. (2020). Biomimetic anchoring of Fe_3_O_4_ onto Ti_3_C_2_ MXene for highly efficient removal of organic dyes by Fenton reaction. J. Environ. Chem. Eng..

[19] Wang L., Jiang H., Wang H., Show P.L., Ivanets A., Luo D., Wang C. (2022). MXenes as heterogeneous Fenton-like catalysts for removal of organic pollutants: A review. J. Environ. Chem. Eng..

[20] Wang C., Ye J., Liang L., Cui X., Kong L., Li N., Cheng Z., Peng W., Yan B., Chen G. (2023). Application of MXene-based materials in Fenton-like systems for organic wastewater treatment: A review. Sci. Total Environ..

[21] Cheng X., Zu L., Jiang Y., Shi D., Cai X., Ni Y., Lin S., Qin Y. (2018). A titanium-based photo-Fenton bifunctional catalyst of mp-MXene/TiO_2−x_ nanodots for dramatic enhancement of catalytic efficiency in advanced oxidation processes. Chem. Commun..

[22] Leichtweis J., Silvestri S., Vieira Y., de Lima Burgo T.A., Foletto E.L. (2021). A novel tin ferrite/polymer composite use in photo-Fenton reactions. Int. J. Environ. Sci. Technol..

[23] Soufi A., Hajjaoui H., Elmoubarki R., Abdennouri M., Qourzal S., Barka N. (2022). Heterogeneous Fenton-like degradation of tartrazine using CuFe_2_O_4_ nanoparticles synthesized by sol-gel combustion. Appl. Surf. Sci. Adv..

[24] Cheng S., Zhao S., Xing B., Liu Y., Zhang C., Xia H. (2022). Preparation of magnetic adsorbent-photocatalyst composites for dye removal by synergistic effect of adsorption and photocatalysis. J. Clean. Prod..

[25] Liu N., Huang W., Zhang X., Tang L., Wang L., Wang Y., Wu M. (2018). Ultrathin graphene oxide encapsulated in uniform MIL-88A (Fe) for enhanced visible light-driven photodegradation of RhB. Appl. Catal. B Environ..

[26] Zhang H., Wang L., Chen Q., Li P., Zhou A., Cao X., Hu Q. (2016). Preparation, mechanical and anti-friction performance of MXene/polymer composites. Mater. Des..

[27] Sargazi S., Hajinezhad M.R., Rahdar A., Zafar M.N., Awan A., Baino F. (2021). Assessment of SnFe_2_O_4_ nanoparticles for potential application in theranostics: Synthesis, characterization, in vitro, and in vivo toxicity. Materials.

[28] Allen-Perry K., Straka W., Keith D., Han S., Reynolds L., Gautam B., Autrey D.E. (2021). Tuning the magnetic properties of two-dimensional MXenes by chemical etching. Materials.

[29] Tambe A.B., Arbuj S.S., Umarji G.G., Jawale N.S., Rane S.B., Kulkarni S.K., Kale B.B. (2022). 2D layered MXene/TiO_2_ nano-heterostructures for photocatalytic H_2_ generation. Graphene 2d Mater..

[30] Lu Y., Li D., Liu F. (2022). Characterizing the chemical structure of Ti_3_C_2_Tx MXene by angle-resolved XPS combined with argon ion etching. Materials.

[31] Jadhav S., Pham H.D., Padwal C., Chougale M., Brown C., Motta N., Ostrikov K., Bae J., Dubal D. (2022). Enhancing mechanical energy transfer of piezoelectric supercapacitors. Adv. Mater. Technol..

[32] El-Monaem E.M.A., Eltaweil A.S., El-Subruiti G.M., Mohy-Eldin M.S., Omer A.M. (2023). Adsorption of nitrophenol onto a novel Fe_3_O_4_-κ-carrageenan/MIL-125 (Ti) composite: Process optimization, isotherms, kinetics, and mechanism. Environ. Sci. Pollut. Res..

[33] Xia W., Wang H., Zeng X., Han J., Zhu J., Zhou M., Wu S. (2014). High-efficiency photocatalytic activity of type II SnO/Sn_3_O_4_ heterostructures via interfacial charge transfer. CrystEngComm.

[34] Eltaweil A.S., Bakr S.S., El-Monaem E.M.A., El-Subruiti G.M. (2023). Magnetic hierarchical flower-like Fe_3_O_4_@ ZIF-67/CuNiMn-LDH catalyst with enhanced redox cycle for Fenton-like degradation of Congo red: Optimization and mechanism. Environ. Sci. Pollut. Res..

[35] Youssef N.A., Shaban S.A., Ibrahim F.A., Mahmoud A.S. (2016). Degradation of methyl orange using Fenton catalytic reaction. Egypt. J. Pet..

[36] Rózsa G., Náfrádi M., Alapi T., Schrantz K., Szabó L., Wojnárovits L., Takács E., Tungler A. (2019). Photocatalytic, photolytic and radiolytic elimination of imidacloprid from aqueous solution: Reaction mechanism, efficiency and economic considerations. Appl. Catal. B Environ..

[37] Wu Y., Li X., Zhao H., Yao F., Cao J., Chen Z., Ma F., Wang D., Yang Q. (2022). 2D/2D FeNi-layered double hydroxide/bimetal-MOFs nanosheets for enhanced photo-Fenton degradation of antibiotics: Performance and synergetic degradation mechanism. Chemosphere.

[38] Song H., Zu D., Li C., Zhou R., Wang Y., Zhang W., Pan S., Cai Y., Li Z., Shen Y. (2021). Ultrafast activation of peroxymonosulfate by reduction of trace Fe^3+^ with Ti_3_C_2_ MXene under neutral and alkaline conditions: Reducibility and confinement effect. Chem. Eng. J..

