# Supplementary material for: Enhanced Redox Cycle of Rod-Shaped MIL-88A/SnFe2O4@MXene Sheets for Fenton-like Degradation of Congo Red: Optimization and Mechanism
Source: Nanomaterials (Basel). 2023 Dec 24;14(1):54. doi: 10.3390/nano14010054 (PMC10780543; doi:10.3390/nano14010054)

Enhanced Redox Cycle of Rod-Shaped MIL-88A/$SnFe_2O_4$@MXene Sheets for Fenton-like Degradation of Congo Red; Optimization and Mechanism

Eman M. Abd El-Monaem [1], Nouf Al Harby [2,*] , Mervette El Batouti [1] and Abdelazeem S. Eltaweil [1,3]

1. Department of Chemistry, Faculty of Science, Alexandria University, Alexandria 21934, Egypt; emanabdelmonaem5925@yahoo.com (E.M.A.E.-M.); mervette.elbatouti@alexu.edu.eg (M.E.B.); abdelazeemeltaweil@alexu.edu.eg (A.S.E.)
2. Department of Chemistry, College of Science, Qassim University, Buraidah 51452, Saudi Arabia
3. Department of Engineering, College of Engineering and Technology, University of Technology and Applied Sciences, Al Khuwair, Muscat P.O. Box 74, Oman

* Correspondence: hrbien@qu.edu.sa

Text S1. The used chemicals in the preparation of MIL-88A/$SnFe_2O_4$@MXene composite.

Tin chloride ($SnCl_2$), ferric chloride hexahydrate ($FeCl_3.6H_2O$), and sodium hydroxide (NaOH) were purchased from Sinopharm Chemical Reagent. Fumaric acid (FA), Ti_3AlC_3 and Sodium thiosulfate pentahydrate ($Na_2S_2O_3 \cdot 5H_2O$) were brought from Guangdong Guanghua Science and Technology. Hydrofluoric acid (HF), and ethanol (C_2H_5OH) were obtained from Alpha Chemika.

Text S2. Characterization tools

The morphology, chemical composition, magnetism and surface charge of MIL-88A, MXene, $SnFe_2O_4$, and MIL-88A/$SnFe_2O_4$@MXene were investigated using Fourier Transform Infrared (Frontier, PerkinElmer, FTIR), Scanning Electron Microscope (JEOL 7500F, SEM), X-Ray Photoelectron Spectroscopy (ESCALAB 250XI, XPS), Zeta Potential (Malvern, ZP), X-Ray Diffraction (PANalytical, XRD), ICP spectroscopy (ICP-OES; Agilent 5110), and Gas chromatography–mass spectrometry (SHIMADZU, GCMS-QP 2010 Ultra, GC-MS).

Figure S1. GC-MS of CR molecules after the Fenton-like degradation by MIL-88A/$SnFe_2O_4$@MXene.

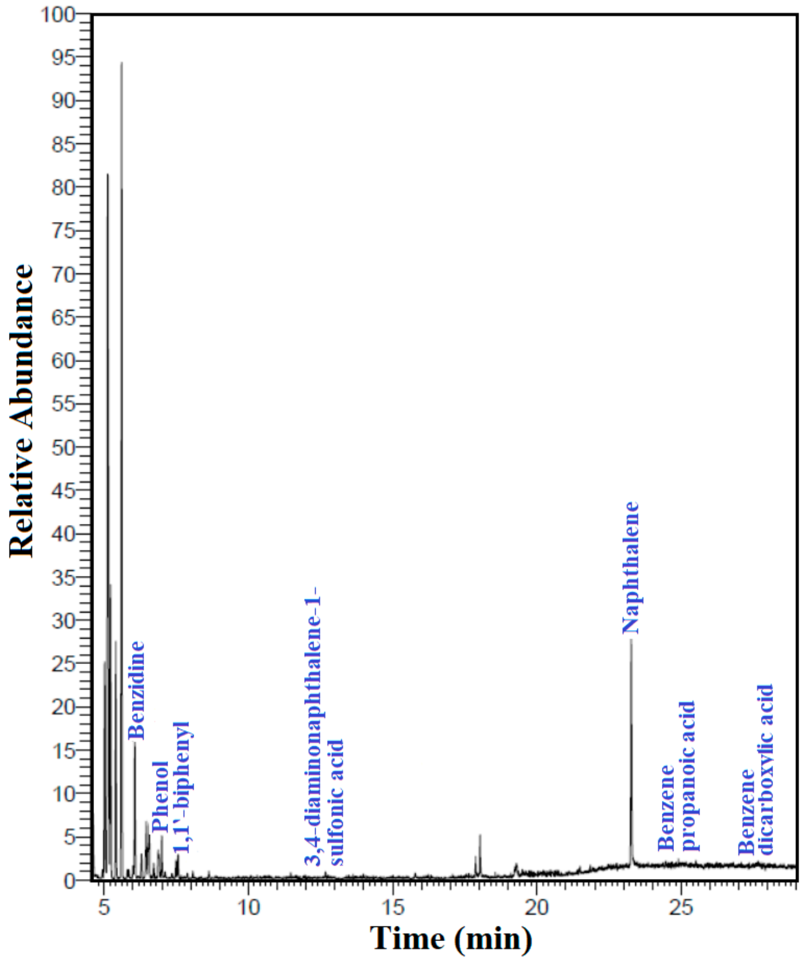

Supplement: Supplementary file 1 [file nanomaterials-14-00054-s001.zip › nanomaterials-2746688-supplementary.pdf]
